# 2-Amino-5-nitro­pyridinium tri­fluoro­acetate

**DOI:** 10.1107/S1600536813011896

**Published:** 2013-05-11

**Authors:** Josephine Viavulamary Jovita, Shanmugam Sathya, Govindasamy Usha, Ramachandran Vasanthi, Krishnan Sarada Ezhilarasi

**Affiliations:** a Postgraduate and Research Department of Physics, Queen Mary’s College, Chennai-4, Tamilnadu, India

## Abstract

The title salt, C_5_H_6_N_3_O_2_^+^·C_2_F_3_O_2_^−^, crystallizes with two cations and two anions in the asymmetric unit. In the crystal, the acetate and pyridine groups are linked by a pair of N—H⋯O hydrogen bonds, forming loops described by the graph-set motif *R*_2_^2^(8). These loops are linked *via* N—H⋯O hydrogen bonds, forming chains along [001]. The chains are in turn linked by C—H⋯O and C—H⋯F hydrogen bonds, generating a three-dimensional supra­molecular network. In both anions, the O and F atoms are disordered over two sites, with occupancy ratios of 0.852 (3):0.148 (3) and 0.851 (3):0.149 (3).

## Related literature

For the biological properties of pyridine derivatives and compounds containing the imidazo[1,2-*a*]pyridine ring system, see: Trapani *et al.* (2003 ▶); Gueiffier *et al.* (1998 ▶); Rival *et al.* (1992 ▶); Rupert *et al.* (2003 ▶). For the crystal structure of a related compound, see: Hemamalini & Fun (2010 ▶). For hydrogen-bond motifs, see: Bernstein *et al.* (1995 ▶).
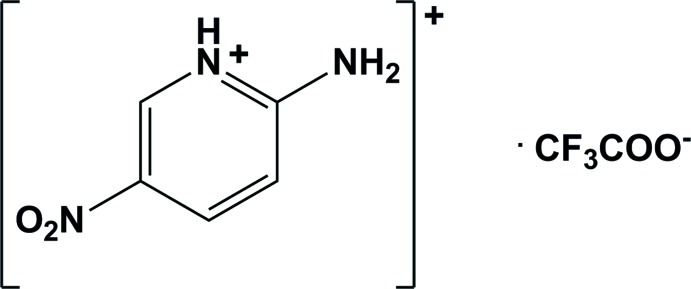


## Experimental

### 

#### Crystal data

C_5_H_6_N_3_O_2_^+^·C_2_F_3_O_2_^−^
*M* *_r_* = 506.30
Monoclinic, 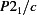

*a* = 19.1388 (7) Å
*b* = 10.7716 (4) Å
*c* = 10.0707 (3) Å
β = 104.668 (2)°
*V* = 2008.47 (12) Å^3^
*Z* = 4
Mo *K*α radiation
μ = 0.17 mm^−1^
*T* = 293 K
0.30 × 0.30 × 0.25 mm

#### Data collection

Bruker APEXII Kappa CCD diffractometer
Absorption correction: multi-scan (*SADABS*; Bruker, 2004 ▶) *T*_min_ = 0.950, *T*_max_ = 0.958
18608 measured reflections
4064 independent reflections
3006 reflections with *I* > 2σ(*I*)
*R*_int_ = 0.027

#### Refinement

*R*[*F* ^2^ > 2σ(*F* ^2^)] = 0.042
*wR*(*F* ^2^) = 0.118
*S* = 1.06
4064 reflections
367 parameters
25 restraints
H atoms treated by a mixture of independent and constrained refinement
Δρ_max_ = 0.28 e Å^−3^
Δρ_min_ = −0.30 e Å^−3^

### 

Data collection: *APEX2* (Bruker, 2004 ▶); cell refinement: *APEX2* and *SAINT* (Bruker, 2004 ▶); data reduction: *SAINT* and *XPREP* (Bruker, 2004 ▶); program(s) used to solve structure: *SIR92* (Altomare *et al.*, 1994 ▶); program(s) used to refine structure: *SHELXL97* (Sheldrick, 2008 ▶); molecular graphics: *PLATON* (Spek, 2009 ▶); software used to prepare material for publication: *SHELXL97*.

## Supplementary Material

Crystal structure: contains datablock(s) I, global. DOI: 10.1107/S1600536813011896/su2582sup1.cif

Structure factors: contains datablock(s) I. DOI: 10.1107/S1600536813011896/su2582Isup2.hkl

Supplementary material file. DOI: 10.1107/S1600536813011896/su2582Isup3.cml

Additional supplementary materials:  crystallographic information; 3D view; checkCIF report

## Figures and Tables

**Table 1:** Hydrogen-bond geometry (Å, °)

*D*—H⋯*A*	*D*—H	H⋯*A*	*D*⋯*A*	*D*—H⋯*A*
N1—H1*A*⋯O4′^i^	0.86	1.90	2.758 (12)	175
N2—H2*A*⋯O3′^i^	0.93 (2)	1.88 (2)	2.791 (4)	168 (2)
N2—H2*B*⋯O8^ii^	0.90 (2)	2.06 (2)	2.935 (7)	166 (2)
N4—H4*A*⋯O8^iii^	0.86	1.91	2.772 (7)	175
N5—H5*A*⋯O7^iii^	0.91 (2)	1.86 (2)	2.763 (9)	172 (2)
N5—H5*B*⋯O3′^iv^	0.87 (2)	1.97 (2)	2.801 (3)	160 (2)
C1—H1⋯O7^v^	0.93	2.36	3.140 (5)	141
C4—H4⋯O2^iv^	0.93	2.57	3.264 (3)	132
C8—H8⋯F1′^vi^	0.93	2.44	3.089 (3)	127
C8—H8⋯O4′^vi^	0.93	2.36	3.253 (7)	161

Symmetry codes: (i) 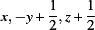; (ii) 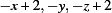; (iii) 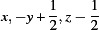; (iv) 
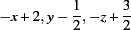
; (v) 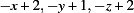; (vi) 
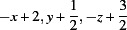
.

**Fig. 1.**
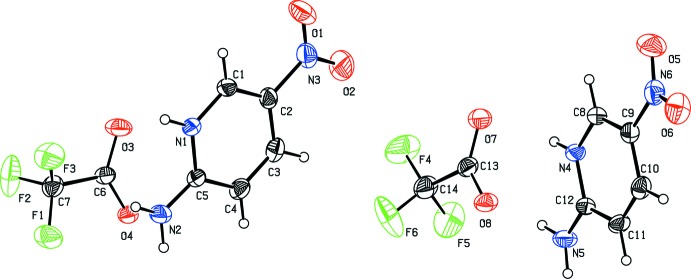
The molecular structure of the title compound, with atom labelling. The displacement ellipsoids are drawn at the 30% probability level. (Only the major components of the disorderd trifluoroacetate anions are shown).

**Fig. 2.**
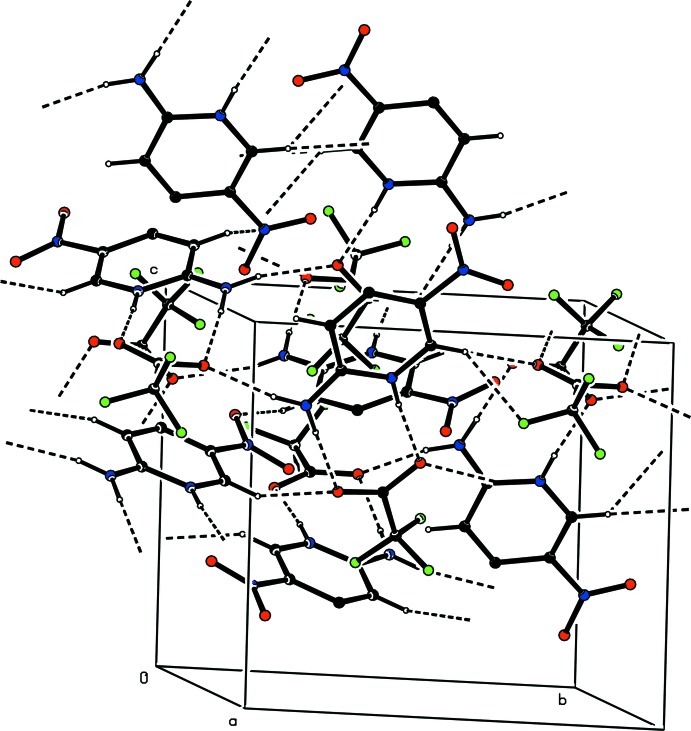
A view along the a axis of the crystal packing of the title compound. The various hydrogen bonds are shown as dashed lines (see Table 1 for details).

## supplementary crystallographic information

### Comment

Pyridine derivatives are important intermediates in organic synthesis, particularly in the synthesis of biologically active and medicinally important agents, for example, in the synthesis of anticonvulsant agents (Trapani *et al.*, 2003) and antiviral agents (Gueiffier *et al.*,1998). Compounds containing the imidazo[1,2-*a*]pyridine ring system have been shown to exhibit antibacterial (Rival *et al.*, 1992) and anti-inflammatory properties (Rupert *et al.*, 2003). The wide spectrum of medicinal applications of this class of compounds prompted us to work in this domain and we report herein on the synthesis and crystal structure of the title compound.

The asymmetric unit of the title compound contains two 2-amino-5-nitro-pyridinium cations and two trifluoroacetate anions, Fig. 1. Each cation is planar with a maximum deviation of -0.0379 (2) Å for atom N3 in cation (C1—C5/N1), and 0.0661 (2) Å for atom N6 in cation (C8—C12/N4). The bond distances in the cations are in good agreement with the values reported for the related structure, 2-amino-5-chloropyridinium trifluoroacetate (Hemamalini & Fun, 2010). The sums of the angles around atoms N1 (360°) and N4 (360°) are an indication of their *sp*^3^ hybridization.

In the crystal, the acetate group and pyridine moiety are linked by a pair of N—H···O hydrogen bonds bonds, forming a loop described by the graph-set motif R^2^_2_(8) (Bernstein *et al.*, 1995). These loops are linked by N—H···O hydrogen bonds to form chains along the b axis (Table 1 and Fig. 2). The chains are further linked by C—H···O and C—H···F hydrogen bonds, generating a three-dimensional supramolecular network.

### Experimental

The title compound was synthesized by the reaction of an equimolar ratio of 2-amino-5-nitropyridine and trifluoroacetic acid. Trifluoroacetic acid was diluted with Millipore water and to this 2-amino-5-nitropyridine was added at room temperature. The mixture was stirred for 6 h to give a homogeneous solution, which was filtered and the filtrate kept for slow evaporation at room temperature. A saturated solution of the solid obtained was prepared by using methanol. It was kept in a constant-temperature water bath at 303 K to stabilize the temperature and avoid the effect of fluctuation in room temperature. After slow evaporation over a period of 15 d large colourless block-like crystals of the title compound were obtained.

### Refinement

The N—H distances of NH_2_ groups were restrained to be equal and were freely refined. The other H atoms were positioned geometrically and treated as riding on their parent atoms: C—H = 0.93 Å, N—H = 0.86 Å, with *U*_iso_(H) = 1.2Ueq(N,C). The F and O atoms of both anions are disordered over two positions with occupancy ratios of 0.852 (3):0.148 (3) and 0.851 (3):0.149 (3).

### Crystal data

**Table:**

C_5_H_6_N_3_O_2_^+^·C_2_F_3_O_2_^−^	*F*(000) = 1024
*M**_r_* = 506.30	*D*_x_ = 1.674 Mg m^−^^3^
Monoclinic, *P*2_1_/*c*	Mo *K*α radiation, λ = 0.71073 Å
Hall symbol: -P 2ybc	Cell parameters from 6393 reflections
*a* = 19.1388 (7) Å	θ = 2.2–25.4°
*b* = 10.7716 (4) Å	µ = 0.17 mm^−^^1^
*c* = 10.0707 (3) Å	*T* = 293 K
β = 104.668 (2)°	Block, colourless
*V* = 2008.47 (12) Å^3^	0.30 × 0.30 × 0.25 mm
*Z* = 4	

### Data collection

**Table:**

Bruker APEXII Kappa CCD diffractometer	4064 independent reflections
Radiation source: fine-focus sealed tube	3006 reflections with *I* > 2σ(*I*)
Graphite monochromator	*R*_int_ = 0.027
ω and φ scans	θ_max_ = 26.3°, θ_min_ = 2.2°
Absorption correction: multi-scan (*SADABS*; Bruker, 2004)	*h* = −23→23
*T*_min_ = 0.950, *T*_max_ = 0.958	*k* = −13→13
18608 measured reflections	*l* = −12→8

### Refinement

**Table:**

Refinement on *F*^2^	Primary atom site location: structure-invariant direct methods
Least-squares matrix: full	Secondary atom site location: difference Fourier map
*R*[*F*^2^ > 2σ(*F*^2^)] = 0.042	Hydrogen site location: inferred from neighbouring sites
*wR*(*F*^2^) = 0.118	H atoms treated by a mixture of independent and constrained refinement
*S* = 1.06	*w* = 1/[σ^2^(*F*_o_^2^) + (0.0512*P*)^2^ + 0.6997*P*] where *P* = (*F*_o_^2^ + 2*F*_c_^2^)/3
4064 reflections	(Δ/σ)_max_ = 0.001
367 parameters	Δρ_max_ = 0.28 e Å^−^^3^
25 restraints	Δρ_min_ = −0.30 e Å^−^^3^

### Special details

**Table:**

Geometry. Bond distances, angles etc. have been calculated using the rounded fractional coordinates. All su's are estimated from the variances of the (full) variance-covariance matrix. The cell esds are taken into account in the estimation of distances, angles and torsion angles.
Refinement. Refinement of F^2^ against ALL reflections. The weighted R-factor wR and goodness of fit S are based on F^2^, conventional R-factors R are based on F, with F set to zero for negative F^2^. The threshold expression of F^2^ > 2sigma(F^2^) is used only for calculating R-factors(gt) etc. and is not relevant to the choice of reflections for refinement. R-factors based on F^2^ are statistically about twice as large as those based on F, and R-factors based on ALL data will be even larger.

### Fractional atomic coordinates and isotropic or equivalent isotropic displacement parameters (Å^2^)

**Table:**

	*x*	*y*	*z*	*U*_iso_*/*U*_eq_	Occ. (<1)
F1'	0.6216 (3)	0.0649 (2)	0.6851 (3)	0.0814 (9)	0.852 (3)
F2'	0.56447 (14)	0.2115 (4)	0.5636 (3)	0.1083 (15)	0.852 (3)
F3'	0.63061 (13)	0.2483 (2)	0.7641 (2)	0.0768 (9)	0.852 (3)
O3'	0.70962 (17)	0.31733 (18)	0.5942 (3)	0.0575 (10)	0.852 (3)
O4'	0.7170 (6)	0.1168 (5)	0.5457 (14)	0.0457 (12)	0.852 (3)
C6	0.69027 (10)	0.20703 (17)	0.58967 (18)	0.0399 (6)	
C7	0.62582 (11)	0.1822 (2)	0.6500 (2)	0.0512 (7)	
F1	0.624 (2)	0.0769 (12)	0.717 (2)	0.0814 (9)	0.148 (3)
F2	0.5709 (9)	0.171 (3)	0.5411 (17)	0.1083 (15)	0.148 (3)
F3	0.6058 (9)	0.2704 (12)	0.7193 (13)	0.0768 (9)	0.148 (3)
O3	0.6905 (13)	0.3188 (9)	0.558 (2)	0.0575 (10)	0.148 (3)
O4	0.725 (4)	0.122 (3)	0.555 (9)	0.0457 (12)	0.148 (3)
F4	1.09841 (15)	0.2673 (2)	0.9010 (3)	0.1233 (10)	0.851 (3)
F5	1.15881 (12)	0.1049 (2)	0.8967 (2)	0.1058 (9)	0.851 (3)
F6	1.08797 (13)	0.1125 (3)	1.0222 (3)	0.1281 (12)	0.851 (3)
O7	1.2114 (4)	0.3372 (2)	1.0927 (10)	0.0567 (16)	0.851 (3)
O8	1.2245 (3)	0.1454 (6)	1.1793 (7)	0.0529 (13)	0.851 (3)
C13	1.19568 (11)	0.22682 (19)	1.0956 (2)	0.0450 (7)	
C14	1.13362 (13)	0.1801 (2)	0.9792 (2)	0.0613 (8)	
F4'	1.1391 (8)	0.2274 (13)	0.8650 (10)	0.1233 (10)	0.149 (3)
F5'	1.1264 (8)	0.0609 (8)	0.9603 (13)	0.1058 (9)	0.149 (3)
F6'	1.0714 (5)	0.2141 (16)	0.9973 (18)	0.1281 (12)	0.149 (3)
O7'	1.195 (2)	0.3400 (9)	1.109 (6)	0.054 (9)	0.149 (3)
O8'	1.2391 (18)	0.150 (3)	1.163 (4)	0.045 (6)	0.149 (3)
O1	0.96571 (10)	0.58183 (17)	0.87250 (19)	0.0753 (7)	
O2	1.00888 (11)	0.4598 (2)	0.7436 (3)	0.0991 (9)	
N1	0.82378 (8)	0.31869 (13)	0.91912 (15)	0.0381 (5)	
N2	0.78170 (10)	0.11850 (16)	0.8954 (2)	0.0517 (6)	
N3	0.96796 (10)	0.4827 (2)	0.8153 (2)	0.0606 (7)	
C1	0.86882 (10)	0.40934 (17)	0.90128 (18)	0.0408 (6)	
C2	0.91883 (10)	0.38461 (18)	0.83148 (19)	0.0437 (6)	
C3	0.92425 (11)	0.2660 (2)	0.7785 (2)	0.0500 (7)	
C4	0.87916 (10)	0.1762 (2)	0.7980 (2)	0.0490 (7)	
C5	0.82704 (9)	0.20153 (17)	0.87184 (18)	0.0385 (5)	
O5	1.44036 (10)	0.50593 (17)	1.1714 (2)	0.0860 (7)	
O6	1.48351 (9)	0.36088 (19)	1.31572 (16)	0.0756 (6)	
N4	1.32454 (8)	0.25957 (13)	0.90524 (15)	0.0403 (5)	
N5	1.30568 (10)	0.06118 (17)	0.82079 (19)	0.0533 (6)	
N6	1.44654 (9)	0.3977 (2)	1.20577 (19)	0.0564 (7)	
C8	1.35833 (10)	0.34404 (17)	0.99745 (19)	0.0417 (6)	
C9	1.40837 (10)	0.30535 (18)	1.10970 (19)	0.0425 (6)	
C10	1.42431 (10)	0.17906 (19)	1.1319 (2)	0.0477 (6)	
C11	1.39007 (11)	0.09627 (18)	1.0385 (2)	0.0459 (6)	
C12	1.33876 (10)	0.13646 (16)	0.91913 (19)	0.0394 (6)	
H1	0.86560	0.48830	0.93670	0.0490*	
H1A	0.79150	0.33570	0.96230	0.0460*	
H2A	0.7521 (11)	0.135 (2)	0.953 (2)	0.065 (7)*	
H2B	0.7837 (13)	0.0419 (17)	0.862 (2)	0.068 (7)*	
H3	0.95870	0.24950	0.73020	0.0600*	
H4	0.88220	0.09710	0.76280	0.0590*	
H4A	1.29250	0.28480	0.83420	0.0480*	
H5A	1.2713 (10)	0.092 (2)	0.7491 (18)	0.065 (7)*	
H5B	1.3104 (13)	−0.018 (2)	0.840 (2)	0.066 (7)*	
H8	1.34740	0.42800	0.98410	0.0500*	
H10	1.45820	0.15290	1.21030	0.0570*	
H11	1.40010	0.01210	1.05240	0.0550*	

### Atomic displacement parameters (Å^2^)

**Table:**

	*U*^11^	*U*^22^	*U*^33^	*U*^12^	*U*^13^	*U*^23^
F1'	0.1041 (13)	0.0575 (10)	0.108 (2)	−0.0127 (10)	0.074 (2)	0.0002 (11)
F2'	0.0443 (10)	0.182 (4)	0.0999 (15)	0.0261 (13)	0.0206 (10)	0.0242 (15)
F3'	0.0902 (18)	0.0800 (13)	0.0738 (14)	0.0095 (10)	0.0457 (12)	−0.0166 (11)
O3'	0.076 (2)	0.0308 (8)	0.076 (2)	−0.0038 (9)	0.0383 (15)	−0.0069 (8)
O4'	0.048 (3)	0.0320 (9)	0.064 (2)	0.0004 (12)	0.027 (3)	−0.0028 (9)
C6	0.0466 (10)	0.0315 (10)	0.0434 (10)	0.0018 (8)	0.0145 (8)	0.0004 (8)
C7	0.0504 (12)	0.0497 (12)	0.0588 (12)	0.0055 (10)	0.0237 (10)	0.0010 (10)
F1	0.1041 (13)	0.0575 (10)	0.108 (2)	−0.0127 (10)	0.074 (2)	0.0002 (11)
F2	0.0443 (10)	0.182 (4)	0.0999 (15)	0.0261 (13)	0.0206 (10)	0.0242 (15)
F3	0.0902 (18)	0.0800 (13)	0.0738 (14)	0.0095 (10)	0.0457 (12)	−0.0166 (11)
O3	0.076 (2)	0.0308 (8)	0.076 (2)	−0.0038 (9)	0.0383 (15)	−0.0069 (8)
O4	0.048 (3)	0.0320 (9)	0.064 (2)	0.0004 (12)	0.027 (3)	−0.0028 (9)
F4	0.109 (2)	0.0852 (15)	0.1244 (18)	0.0264 (13)	−0.0654 (16)	0.0034 (13)
F5	0.0952 (16)	0.1350 (19)	0.0739 (13)	0.0144 (14)	−0.0033 (10)	−0.0568 (13)
F6	0.0820 (14)	0.178 (3)	0.1117 (17)	−0.0743 (18)	0.0010 (13)	−0.0013 (19)
O7	0.056 (4)	0.0419 (17)	0.0666 (17)	0.0009 (13)	0.005 (2)	0.0002 (15)
O8	0.056 (3)	0.0368 (15)	0.055 (2)	0.0024 (17)	−0.006 (2)	−0.0057 (13)
C13	0.0423 (11)	0.0408 (12)	0.0486 (11)	0.0064 (9)	0.0052 (8)	−0.0068 (9)
C14	0.0556 (13)	0.0542 (14)	0.0627 (14)	0.0072 (11)	−0.0061 (11)	−0.0064 (11)
F4'	0.109 (2)	0.0852 (15)	0.1244 (18)	0.0264 (13)	−0.0654 (16)	0.0034 (13)
F5'	0.0952 (16)	0.1350 (19)	0.0739 (13)	0.0144 (14)	−0.0033 (10)	−0.0568 (13)
F6'	0.0820 (14)	0.178 (3)	0.1117 (17)	−0.0743 (18)	0.0010 (13)	−0.0013 (19)
O7'	0.043 (14)	0.026 (8)	0.08 (2)	0.013 (6)	−0.006 (12)	−0.014 (8)
O8'	0.034 (9)	0.044 (10)	0.059 (10)	0.005 (7)	0.015 (7)	−0.007 (6)
O1	0.0771 (12)	0.0604 (11)	0.0829 (12)	−0.0246 (9)	0.0099 (9)	0.0145 (9)
O2	0.0768 (13)	0.0950 (15)	0.1466 (19)	0.0023 (11)	0.0674 (14)	0.0403 (13)
N1	0.0381 (8)	0.0327 (8)	0.0460 (8)	0.0026 (6)	0.0153 (6)	−0.0017 (6)
N2	0.0548 (11)	0.0353 (10)	0.0711 (12)	−0.0039 (8)	0.0271 (9)	−0.0098 (8)
N3	0.0436 (10)	0.0655 (14)	0.0702 (12)	−0.0022 (9)	0.0098 (9)	0.0302 (11)
C1	0.0414 (10)	0.0332 (9)	0.0453 (10)	0.0011 (8)	0.0062 (8)	0.0046 (8)
C2	0.0334 (9)	0.0471 (11)	0.0488 (10)	0.0008 (8)	0.0069 (8)	0.0134 (9)
C3	0.0377 (10)	0.0625 (14)	0.0528 (11)	0.0094 (9)	0.0169 (9)	0.0043 (10)
C4	0.0451 (11)	0.0465 (12)	0.0564 (11)	0.0077 (9)	0.0147 (9)	−0.0095 (9)
C5	0.0369 (9)	0.0337 (9)	0.0440 (9)	0.0035 (8)	0.0085 (8)	−0.0022 (8)
O5	0.0863 (13)	0.0543 (11)	0.1018 (14)	−0.0114 (10)	−0.0050 (11)	−0.0169 (10)
O6	0.0656 (10)	0.1026 (14)	0.0505 (9)	0.0010 (10)	−0.0002 (8)	−0.0128 (9)
N4	0.0404 (8)	0.0309 (8)	0.0449 (8)	0.0026 (6)	0.0024 (7)	0.0069 (7)
N5	0.0614 (11)	0.0309 (9)	0.0612 (11)	−0.0006 (8)	0.0036 (9)	0.0047 (8)
N6	0.0433 (10)	0.0675 (13)	0.0565 (11)	−0.0012 (9)	0.0093 (8)	−0.0115 (9)
C8	0.0407 (10)	0.0317 (9)	0.0524 (10)	0.0002 (8)	0.0115 (8)	0.0022 (8)
C9	0.0368 (10)	0.0452 (11)	0.0456 (10)	0.0002 (8)	0.0107 (8)	−0.0004 (8)
C10	0.0424 (10)	0.0550 (13)	0.0444 (10)	0.0077 (9)	0.0087 (8)	0.0127 (9)
C11	0.0501 (11)	0.0354 (10)	0.0522 (11)	0.0088 (9)	0.0129 (9)	0.0142 (9)
C12	0.0398 (10)	0.0305 (9)	0.0489 (10)	0.0007 (8)	0.0131 (8)	0.0075 (8)

### Geometric parameters (Å, º)

**Table:**

F1—C7	1.325 (16)	N3—C2	1.451 (3)
F1'—C7	1.320 (3)	N1—H1A	0.8600
F2—C7	1.319 (17)	N2—H2B	0.896 (19)
F2'—C7	1.311 (4)	N2—H2A	0.93 (2)
F3—C7	1.293 (14)	N4—C12	1.354 (2)
F3'—C7	1.335 (3)	N4—C8	1.343 (2)
F4—C14	1.299 (3)	N5—C12	1.312 (3)
F4'—C14	1.286 (11)	N6—C9	1.450 (3)
F5—C14	1.335 (3)	N4—H4A	0.8600
F5'—C14	1.300 (9)	N5—H5A	0.908 (19)
F6—C14	1.293 (4)	N5—H5B	0.87 (2)
F6'—C14	1.302 (12)	C6—C7	1.530 (3)
O3—C6	1.246 (11)	C13—C14	1.527 (3)
O3'—C6	1.242 (3)	C1—C2	1.350 (3)
O4—C6	1.23 (6)	C2—C3	1.399 (3)
O4'—C6	1.232 (9)	C3—C4	1.343 (3)
O7—C13	1.229 (4)	C4—C5	1.414 (3)
O7'—C13	1.227 (12)	C1—H1	0.9300
O8—C13	1.244 (7)	C3—H3	0.9300
O8'—C13	1.25 (4)	C4—H4	0.9300
O1—N3	1.219 (3)	C8—C9	1.349 (3)
O2—N3	1.217 (3)	C9—C10	1.400 (3)
O5—N6	1.213 (3)	C10—C11	1.341 (3)
O6—N6	1.220 (2)	C11—C12	1.414 (3)
N1—C1	1.344 (2)	C8—H8	0.9300
N1—C5	1.356 (2)	C10—H10	0.9300
N2—C5	1.309 (3)	C11—H11	0.9300
			
C1—N1—C5	122.76 (16)	F4—C14—C13	114.2 (2)
O1—N3—C2	118.91 (19)	F5'—C14—C13	118.1 (6)
O2—N3—C2	116.9 (2)	F6'—C14—C13	111.2 (8)
O1—N3—O2	124.2 (2)	F4'—C14—F5'	106.9 (8)
C5—N1—H1A	119.00	F5—C14—C13	110.2 (2)
C1—N1—H1A	119.00	F4'—C14—C13	109.4 (6)
H2A—N2—H2B	120 (2)	F5—C14—F6	104.4 (2)
C5—N2—H2B	117.9 (16)	F4—C14—F6	108.6 (3)
C5—N2—H2A	121.5 (13)	F5'—C14—F6'	103.4 (10)
C8—N4—C12	122.89 (16)	F6—C14—C13	112.9 (2)
O5—N6—C9	118.21 (18)	F4'—C14—F6'	107.4 (10)
O6—N6—C9	117.5 (2)	F4—C14—F5	106.0 (2)
O5—N6—O6	124.3 (2)	N1—C1—C2	119.31 (17)
C12—N4—H4A	119.00	N3—C2—C3	120.64 (18)
C8—N4—H4A	119.00	C1—C2—C3	120.66 (18)
C12—N5—H5B	115.7 (14)	N3—C2—C1	118.69 (18)
C12—N5—H5A	119.2 (13)	C2—C3—C4	119.31 (19)
H5A—N5—H5B	123 (2)	C3—C4—C5	120.19 (19)
O3'—C6—C7	114.6 (2)	N2—C5—C4	123.50 (18)
O4—C6—C7	122 (3)	N1—C5—N2	118.73 (17)
O4'—C6—C7	117.1 (5)	N1—C5—C4	117.76 (17)
O3—C6—C7	109.0 (11)	C2—C1—H1	120.00
O3'—C6—O4'	128.4 (5)	N1—C1—H1	120.00
O3—C6—O4	128 (3)	C2—C3—H3	120.00
F2'—C7—F3'	107.3 (2)	C4—C3—H3	120.00
F2—C7—F3	103.5 (14)	C5—C4—H4	120.00
F1'—C7—F3'	106.0 (2)	C3—C4—H4	120.00
F1'—C7—C6	112.6 (3)	N4—C8—C9	119.03 (17)
F3'—C7—C6	111.22 (19)	N6—C9—C8	118.56 (18)
F3—C7—C6	117.7 (7)	C8—C9—C10	120.92 (18)
F2'—C7—C6	111.8 (2)	N6—C9—C10	120.51 (17)
F1—C7—F3	107.7 (12)	C9—C10—C11	119.10 (18)
F2—C7—C6	103.8 (8)	C10—C11—C12	120.25 (18)
F1—C7—F2	103.1 (18)	N4—C12—C11	117.77 (16)
F1'—C7—F2'	107.6 (3)	N5—C12—C11	123.44 (17)
F1—C7—C6	118.7 (16)	N4—C12—N5	118.78 (17)
O8'—C13—C14	118.8 (16)	N4—C8—H8	120.00
O7—C13—O8	129.0 (5)	C9—C8—H8	120.00
O7—C13—C14	116.6 (5)	C9—C10—H10	120.00
O7'—C13—O8'	129 (3)	C11—C10—H10	120.00
O7'—C13—C14	113 (2)	C10—C11—H11	120.00
O8—C13—C14	114.4 (3)	C12—C11—H11	120.00
			
C1—N1—C5—C4	−1.6 (3)	O8—C13—C14—F6	47.9 (4)
C1—N1—C5—N2	179.07 (18)	O7—C13—C14—F4	−9.0 (5)
C5—N1—C1—C2	1.2 (3)	O8—C13—C14—F4	172.6 (4)
O2—N3—C2—C1	−174.9 (2)	O8—C13—C14—F5	−68.3 (4)
O1—N3—C2—C1	4.7 (3)	O7—C13—C14—F5	110.1 (5)
O2—N3—C2—C3	6.5 (3)	O7—C13—C14—F6	−133.7 (5)
O1—N3—C2—C3	−173.9 (2)	N1—C1—C2—N3	−178.84 (17)
C12—N4—C8—C9	−0.5 (3)	N1—C1—C2—C3	−0.3 (3)
C8—N4—C12—C11	1.9 (3)	C1—C2—C3—C4	−0.2 (3)
C8—N4—C12—N5	−176.76 (19)	N3—C2—C3—C4	178.37 (19)
O5—N6—C9—C10	167.4 (2)	C2—C3—C4—C5	−0.3 (3)
O5—N6—C9—C8	−11.3 (3)	C3—C4—C5—N1	1.1 (3)
O6—N6—C9—C10	−11.8 (3)	C3—C4—C5—N2	−179.6 (2)
O6—N6—C9—C8	169.47 (19)	N4—C8—C9—C10	−1.1 (3)
O4'—C6—C7—F3'	138.5 (7)	N4—C8—C9—N6	177.59 (17)
O4'—C6—C7—F1'	19.7 (7)	C8—C9—C10—C11	1.2 (3)
O3'—C6—C7—F2'	79.7 (3)	N6—C9—C10—C11	−177.44 (19)
O4'—C6—C7—F2'	−101.6 (7)	C9—C10—C11—C12	0.2 (3)
O3'—C6—C7—F1'	−159.0 (3)	C10—C11—C12—N5	176.9 (2)
O3'—C6—C7—F3'	−40.3 (3)	C10—C11—C12—N4	−1.7 (3)

### Hydrogen-bond geometry (Å, º)

**Table:**

*D*—H···*A*	*D*—H	H···*A*	*D*···*A*	*D*—H···*A*
N1—H1*A*···O4′^i^	0.86	1.90	2.758 (12)	175
N2—H2*A*···O3′^i^	0.93 (2)	1.88 (2)	2.791 (4)	168 (2)
N2—H2*B*···O8^ii^	0.90 (2)	2.06 (2)	2.935 (7)	166 (2)
N4—H4*A*···O8^iii^	0.86	1.91	2.772 (7)	175
N5—H5*A*···O7^iii^	0.91 (2)	1.86 (2)	2.763 (9)	172 (2)
N5—H5*B*···O3′^iv^	0.87 (2)	1.97 (2)	2.801 (3)	160 (2)
C1—H1···O7^v^	0.93	2.36	3.140 (5)	141
C4—H4···O2^iv^	0.93	2.57	3.264 (3)	132
C8—H8···F1′^vi^	0.93	2.44	3.089 (3)	127
C8—H8···O4′^vi^	0.93	2.36	3.253 (7)	161

Symmetry codes: (i) *x*, −*y*+1/2, *z*+1/2; (ii) −*x*+2, −*y*, −*z*+2; (iii) *x*, −*y*+1/2, *z*−1/2; (iv) −*x*+2, *y*−1/2, −*z*+3/2; (v) −*x*+2, −*y*+1, −*z*+2; (vi) −*x*+2, *y*+1/2, −*z*+3/2.
